# Estimated incidence of influenza in Guangzhou, China, 2019–2022

**DOI:** 10.1016/j.imj.2025.100221

**Published:** 2025-11-20

**Authors:** Di Wu, Zhaonian Tan, Yanhui Liu, Mengmeng Ma, Zhitao Chen, Dedong Wang, Lei Luo, Pengzhe Qin

**Affiliations:** a Guangzhou Center for Disease Control and Prevention, School of Public Health, Guangzhou Medical University, Guangzhou 510440, Guangdong Province, China; b School of Public Health, Guangdong Pharmaceutical University, Guangzhou 510006, Guangdong Province, China; c Department of Public Health and Preventive Medicine, School of Medicine, Jinan University, Guangzhou 510632, Guangdong Province, China

**Keywords:** Influenza, Infection rate, Incidence rate

## Abstract

**Background:**

Influenza is a significant public health issue, particularly for vulnerable groups like children and the elderly. Despite widespread vaccination and public health measures, age-specific incidence data-crucial for targeted interventions—are limited in many areas, including Guangzhou. The epidemiological patterns of influenza have also been affected by non-pharmaceutical interventions during the COVID-19 pandemic, underscoring the need for updated local estimates. This study aimed to estimate the age-specific incidence of influenza infection in Guangzhou from 2019 to 2022—a period covering both pre-pandemic and pandemic phases—to inform regionally tailored prevention and control strategies.

**Methods:**

This study analyzed surveillance data on influenza-like illness (ILI) and virological test results from sentinel hospitals in Guangzhou covering the period from 2019 to 2022. A previously established multiplier model was employed, which integrated age-specific consultation rates, influenza positivity rates, as well as parameters related to symptom presentation and detection sensitivity. Monte Carlo simulations were utilized to estimate annual age-stratified influenza infection and incidence rates, accompanied by 95% confidence intervals. The population denominators were derived from the national census conducted in 2020.

**Results:**

7.78% of the total population in Guangzhou were infected by influenza in 2019, 1.40% in 2020, 1.85% in 2021 and 12.13% in 2022 and incidence rates were 5.15% in 2019, 0.93% in 2020, 1.23% in 2021 and 8.04% in 2022. The highest influenza infection and incidence rates were observed in 2022 and the lowest in 2020. Infections in the 0–14 age group were 27.19%, 3.57%, 11.16% and 66.15% during 2019–2022 and respective incidence rates were 18.00%, 2.37%, 7.41% and 43.84%.

**Conclusions:**

0–14-year-old infants and children were the main victims of influenza. Targeted strategies should be developed to prevent the spread in this age group.

## Highlights

• Estimation of influenza infection and incidence rates in Guangzhou from 2019 to 2022;
• 0–14 years old infants and children were the main victims of influenza.

## Graphical Abstract

**Figure:**
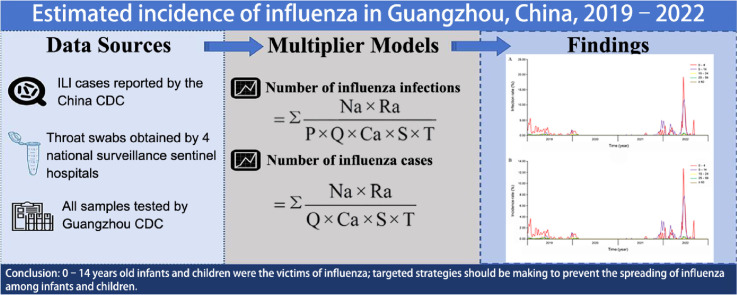

## 1 Introduction

Influenza viruses caused acute respiratory disease with seasonal cycles.^1^. Annual infection rates are 5%–10% adults and 20%–30% adolescents with 3 to 5 million severe cases and 0.29 to 0.65 million seasonal influenza-associated deaths out of 1 billion cases worldwide.^2^^,^^3^ The health and economic burden of influenza-related illness was reported heavy in China, and the mortality of the influenza-associated diseases was estimated as 14.3 per 100,000.^4^ The Chinese Center for Disease Control and Prevention (CDC) had estimated that an average of 88,100 influenza-associated excess respiratory deaths occurred annually in China, which account for 8.2% of all respiratory deaths,^5^ and Beijing CDC has reported that 10.46% of the whole population were reported infected, with an incidence rate of 6.92% during 2017–2018 influenza season in Beijing, China.^6^ Influenza not only affects the health of individuals but also places a heavy financial burden on their families, especially among families with infants, adolescents, and the elderly.^7^

Located in southern China, Guangzhou has a subtropical climate with high humidity, abundant precipitation, and marked seasonal temperature differences. These conditions can sustain influenza virus activity for extended periods, leading to prolonged epidemic seasons. As a densely populated megacity with frequent population movement, Guangzhou presents heightened transmission risk. Studying this setting not only reflects influenza patterns in hot, humid, and densely populated environments but also offers valuable reference for understanding transmission in regions with similar climatic and demographic characteristics.

The twindemic of severe acute respiratory syndrome coronavirus 2 (SARS-COV-2) and influenza in the past 3 years showed a significant increase in the risk of respiratory related death^8^, ^9^, ^10^, especially among those with co-infection of SARS-CoV-2 and influenza.^11^ The measures implemented to fight against the SARS-COV-2 pandemic had a significant effect on the spread of influenza.

The current study aimed to estimate influenza incidence in Guangzhou during 2019–2022 and to analyze the degree to which anti-COVID-19 measures affected influenza transmission. The objective was to inform public health strategies, especially in urban areas, ensuring preparedness for future influenza outbreaks.

## 2 Methods

### 2.1 Data sources

Clinical cases of influenza-like-illness (ILI) were obtained from the China Information System for Disease Control and Prevention. ILI was reported and monitored by the Guangzhou Public Health Event Monitoring and Early Warning System. Pathogenic monitoring was conducted by sampling from four national surveillance sentinel hospitals, namely: Guangzhou First People's Hospital, Liwan Central Hospital of Guangzhou, Guangzhou Red Cross Hospital, and Children's Hospital of Guangzhou Women and Children's Medical Center. Samples were tested by Guangzhou Center for Disease Control and Prevention. The study size was calculated to give a robust dataset for analysis and contained no missing data.

### 2.2 Influenza-like illness surveillance

ILI cases were defined as fever (≥ 38°C), accompanied by cough or sore throat, and were reported by all the municipal surveillance sentinel hospitals in Guangzhou (21 hospitals, including the 4 national surveillance sentinel hospitals). A weekly report was submitted on the number of ILI cases and outpatients grouped by age (0–4, 5–14, 15–24, 25–59 and ≥ 60 years).

### 2.3 Influenza virus surveillance

Each week, 20 throat swabs from outpatients and 5 throat swabs from inpatients diagnosed with severe acute respiratory infection were obtained by the 4 national surveillance sentinel hospitals, and all throat swabs samples were tested by Guangzhou CDC following the manufacturer's guidelines.

### 2.4 Multiplier model

The multiplier model used in this study was used for assessing the infection and incidence rates of seasonal influenza in Beijing from 2015 to 2016. The calculation can be divided into three steps.

Step 1: Numbers of seasonal influenza infections and patients were obtained from the following parameters: (1) *N*a: Reported age-specific number of ILI consultations from ILI surveillance;^12^^,^^13^ (2) *R*a: Age-specific proportion of positive cases from influenza virus surveillance;^12^^,^^13^ (3) *P*: Proportion of dominant infection among influenza infections. Dominant infection was defined as infection with pathological changes and clinical manifestations (range: 58.3%–74.5%)^12^^,^^14^^,^^15^ (4) *Q*: ILI cases as a proportion of symptomatic infections (range: 26.0%–42.0%);^12^^,^^14^ (5) *C*a: Age-specific consultation rates (from the fourth National Health Services Survey of China, 2008, https://www.nhc.gov.cn/cmsresources/mohwsbwstjxxzx/cmsrsdocument/doc9912.pdf) were as follows: 0–4 years: 72.7%; 5–14 years: 59.7%; 15–24 years: 50.5%; 25–59 years: 50.5% and 60 + years: 71.2%; (6) *S*: Success rate of throat swab sampling (range: 80.0%–90.0%);^12^^,^^14^^,^^16^^,^^17^ (7) *T*: Sensitivity of PCR detection (range: 95.0%–100.0%);^12^^,^^14^^,^^16^^,^^17^

Step 2: Previous surveillance data and the above parameters were substituted into formulae (1) and (2) to calculate the numbers of infected and affected individuals in each age group for the period 2019 to 2022:

(1) $$\begin{array}{ccc} & & \text{Total}\;\text{number}\;\text{of}\;\text{influenza}\;\text{infections}\\ & & =\sum \frac{Na\times Ra}{P\times Q\times Ca\times S\times T}\;\;\left[12,14\right] \end{array}$$

(2) $$\begin{array}{ccc} & & \text{Total}\;\text{number}\;\text{of}\;\text{influenza}\;\text{cases}\\ & & =\sum \frac{Na\times Ra}{Q\times Ca\times S\times T}\;\;\left[12,14\right] \end{array}$$

Exact values were used for Ca and other parameters, assumed to obey probability distributions. *R*a was assumed to follow a binomial distribution, while other parameters (*N*a, *Q, S* and *T*) were assumed to follow a uniform distribution since only maximum and minimum values were available. Monte Carlo simulations were conducted with 10,000 iterations of the multiplicative model to compute weekly mean numbers of infections and cases, along with 95% confidence intervals. Weekly numbers of infections and cases were aggregated to obtain annual numbers.^12^

Step 3: Infection and incidence rates were calculated by dividing the numbers of infected people and infected individuals by the population size in each age group in Guangzhou. Population size by age group was derived from the results of the seventh national census (2020).

## 3 Results

### 3.1 Estimated numbers of infection and incidence

The total number of influenza infections in Guangzhou was estimated to be 1,455,347 in 2019, 260,837 in 2020, 344,973 in 2021, and 2,265,501 in 2022. The highest number of infections was recorded in 2022, followed by 2019 and the lowest in 2020. The majority of infections occurred in the age groups 0–4 and 5–14 with the fewest cases among individuals aged over 60 years (Table 1).

**Table 1:** Estimated numbers of influenza infections (2019–2022).

Age group (years)	95% CI			
	2019	2020	2021	2022
0–4	546,912 (538,094–555,731)	57,168 (53,951–60,385)	60,265 (56,262–64,268)	691,676 (659,767–723,585)
5–14	157,427 (150,662–164,192)	35,410 (33,870–36,950)	228,888 (218,385–239,391)	1,021,697 (981,087–1,062,307)
15–24	169,375 (161,554–177,197)	45,497 (43,575–47,418)	2,652 (2,456–2,847)	50,924 (48,638–53,210)
25–59	535,842 (513,146–558,537)	114,482 (109,715–119,250)	51,189 (48,164–54,215)	463,234 (444,043–482,424)
≥ 60	43,790 (41,434–46,146)	8,280 (7,857–8,703)	1,979 (1,833–2,125)	37,969 (36,328–39,611)
Total	1,453,347 (1,404,890–1,501,804)	260,837 (248,969–272,705)	344,973 (327,100–362,846)	2,265,501 (2,169,863–2,361,138)

*Note*: Data are presented as OR (95% CI).

The estimated total numbers of influenza cases were 962,683 in 2019, 172,918 in 2020, 228,847 in 2021, and 1,501,597 in 2022. The highest number of cases was observed in 2022, followed by 2019 and the lowest in 2020. The age groups 0–4 and 5–14 age groups had the highest number of cases with the lowest in those aged 60 years and above (Table 2).

**Table 2:** Estimated numbers of influenza incidences.

Age group (years)	95% CI			
	2019	2020	2021	2022
0–4	362,055 (356,251–367,860)	37,898 (35,775–40,021)	40,015 (37,365–42,665)	458,351 (437,377–479,326)
5–14	104,292 (99,851–108,733)	23,503 (22,485–24,521)	151,822 (144,890–158,753)	677,252 (650,519–703,985)
15–24	112,212 (107,070–117,354)	30,172 (28,906–31,438)	1,759 (1,630–1,888)	33,752 (32,246–35,258)
25–59	355,119 (340,194–370,043)	75,847 (72,716–78,979)	33,937 (31,941–35,934)	307,077 (294,446–319,709)
≥ 60	29,005 (27,454–30,556)	5,497 (5,218–5,777)	1,314 (1,217–1,411)	25,165 (24,086–26,244)
Total	962,683 (930,820–994,546)	172,918 (165,100–180,736)	228,847 (217,043–240,650)	1,501,597 (1,438,674–1,564,521)

*Note*: Data are presented as OR (95% CI).

### 3.2 Estimates of influenza infection and incidence rates

The estimated influenza infection rates in Guangzhou were as follows: 7.78% (95% CI, 7.52%–8.04%) in 2019, 1.40% (95% CI, 1.33%–1.46%) in 2020, 1.85% (95% CI, 1.75%–1.94%) in 2021, and 12.13% (95% CI, 11.62%–12.64%) in 2022. The highest infection rate was observed in 2022, followed by 2019, while the lowest rate was recorded in 2020. Notably, the 0–4 and 5–14 age groups had the highest infection rates, whereas the over 60 group had the lowest rates (Table 3).

**Table 3:** Estimated rates of influenza infections (%).

Age group (years)	95% CI			
	2019	2020	2021	2022
0–4	53.68 (52.82, 54.55)	5.61 (5.30, 5.93)	5.92 (5.52, 6.31)	67.89 (64.76, 71.02)
5–14	10.02 (9.59, 10.45)	2.25 (2.16, 2.35)	14.57 (13.90, 15.23)	65.02 (62.44, 67.60)
15–24	5.84 (5.57, 6.11)	1.57 (1.50, 1.64)	0.09 (0.08, 0.10)	1.76 (1.68, 1.84)
25–59	4.85 (4.64, 5.05)	1.04 (0.99, 1.08)	0.46 (0.44, 0.49)	4.19 (4.02, 4.36)
≥ 60	2.06 (1.94, 2.17)	0.39 (0.37, 0.41)	0.09 (0.09, 0.10)	1.78 (1.71, 1.86)
**0–14**	27.19 (26.59, 27.79)	3.57 (3.39, 3.76)	11.16 (10.60, 11.72)	66.15 (63.35, 68.95)
**≥ 14**	4.66 (4.45, 4.86)	1.05 (1.00, 1.09)	0.35 (0.33, 0.37)	3.43 (3.29, 3.58)
Total	7.78 (7.52, 8.04)	1.40 (1.33, 1.46)	1.85 (1.75, 1.94)	12.13 (11.62, 12.64)

*Note*: Data are presented as OR (95% CI).

In terms of overall influenza incidence, the rates in Guangzhou were 5.15% (95% CI, 4.98%–5.33%) in 2019, 0.93% in 2020 (95% CI, 0.88%–0.97%), 1.23% in 2021 (95% CI, 1.16%–1.29%), and 8.04% in 2022 (95% CI, 7.70%–8.38%). Similar to infection rates, the highest incidence occurred in 2022, followed by 2019, and the lowest incidence was observed in 2020. Specifically, the age groups 0–4 and 5–14 had the highest incidence, while those aged 60 years and older had the lowest incidence rates (Table 4).

**Table 4:** Estimated rates of influenza incidences (%).

Age group (years)	95% CI			
	2019	2020	2021	2022
0–4	35.54 (34.97, 36.11)	3.72 (3.51, 3.93)	3.93 (3.67, 4.19)	44.99 (42.93, 47.05)
5–14	6.64 (6.35, 6.92)	1.50 (1.43, 1.56)	9.66 (9.22, 10.10)	43.10 (41.40, 44.80)
15–24	3.87 (3.69, 4.05)	1.04 (1.00, 1.08)	0.06 (0.06, 0.07)	1.16 (1.11, 1.22)
25–59	3.21 (3.08, 3.35)	0.69 (0.66, 0.71)	0.31 (0.29, 0.32)	2.78 (2.66, 2.89)
≥ 60	1.36 (1.29, 1.43)	0.26 (0.24, 0.27)	0.06 (0.06, 0.07)	1.18 (1.13, 1.23)
**0–14**	18.00 (17.61, 18.40)	2.37 (2.25, 2.49)	7.41 (7.04, 7.78)	43.84 (42.00, 45.68)
**≥ 14**	3.09 (2.95, 3.22)	0.69 (0.66, 0.72)	0.23 (0.22, 0.24)	2.28 (2.18, 2.37)
Total	5.15 (4.98, 5.33)	0.93 (0.88, 0.97)	1.23 (1.16, 1.29)	8.04 (7.70, 8.38)

*Note*: Data are presented as OR (95% CI).

### 3.3 Estimated weekly distribution of influenza infection and incidence rates

**Fig. 1:**
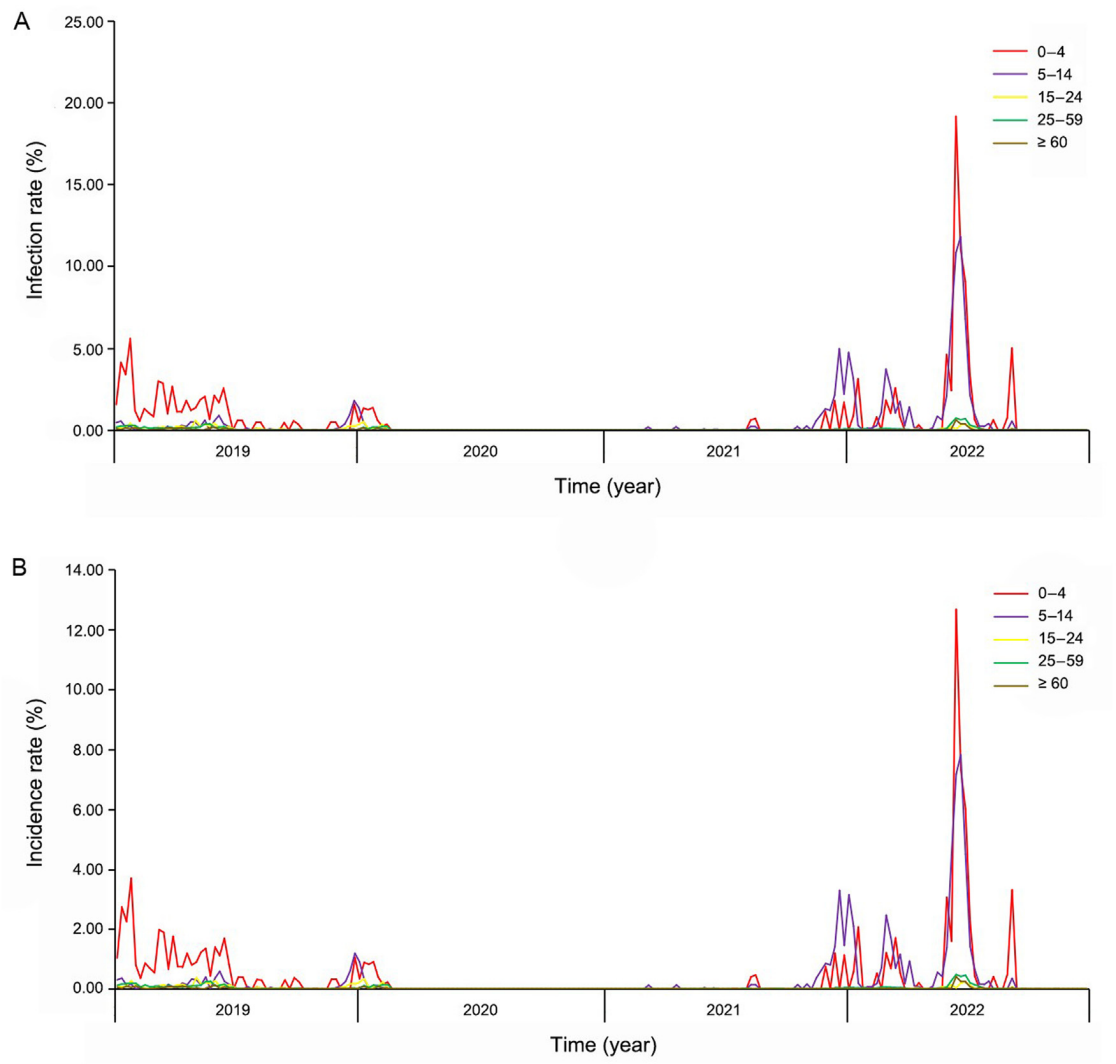
Infection and incidence rates of Guangzhou 2019–2022. (A) Weekly infection rates of Guangzhou 2019–2022. (B) Weekly incidence rates of Guangzhou 2019–2022.

Weekly distributions of estimated influenza infection and incidence rates in Guangzhou from 2019 to 2022 are shown in Fig. 1A and 1B. Considerable fluctuations were found in the age groups 0–4 and 5–14, but mortality and incidence rates remained consistently low in the remaining three age groups.

The rates of infection and incidence among the 0–4 years population increased at the beginning of the year, peaked in the 4th week, decreased and remained constant from the 5th week to the 26th week, further decreased from the 27th week to the 49th week, and showed a slight increase toward the end of the year. A slight peak in infection and incidence was found in the age group 5 to 14 years towards the end of the year, with maximum levels similar to those in the age group 0 to 4. Rates remained around 0% for the rest of the year (Fig. 1).

The chronology of infection and incidence followed a pattern where there was an increase in late 2019 among the 5–14 age group, which declined to 0 around the 4th week of 2020 and remained low until the 45th week of 2021 when a peak occurred. Infection and incidence rates were higher among the 5–14 age group than among the 0–4-years group during this period (Fig. 1). Infection and incidence rates remained around 0 in the population aged 0–4 years except for a tiny peak in early 2020 and a fluctuation at the end of 2021.

The peaks of infection and morbidity in children aged 0 to 4 years occurred during the 3rd, 11th, 24th, and 36th week of 2022, with the highest peak observed in 24th week. Peaks were also observed in the 2nd, 9th, and 26th week in the 5–14 age group, with the highest peak again in 24th week (Fig.1).

## 4 Discussion

According to the World Health Organization, the annual incidence of influenza in children was estimated to be 20%–30% and 5%–10% in adults.^18^^,^^19^ In both 2019 and 2022, the infection and incidence rates were higher in the 0–4 age group than in the 5–14 age group. Previous studies have also reported a heavier disease burden in children aged 0–4 years.^12^^,^^14^ In 2019, people over 60 years old had the lowest infection and incidence rates and the second lowest in 2022. Infants and children were more susceptible due to poor hygiene habits and activities in crowded places like schools while the elderly had a relatively lower infection and incidence rate, possibly due to the lower activity levels and sustained immunity.^20^^,^^21^

Vaccination is the primary strategy for influenza prevention. It reduces individual susceptibility and viral shedding, thereby limiting transmission in high-contact settings such as households and schools. Children are particularly important targets due to their heightened vulnerability and central role in community spread. Nevertheless, the current influenza vaccine coverage in China remains insufficient,^22^^,^^23^ thereby constraining its overall effectiveness at the population level. Increasing vaccination rates, especially among pediatric groups, has the potential to markedly reduce influenza incidence and mitigate the risk of severe outcomes associated with infection.

Estimated influenza infection and incidence rates were lower during 2020 and 2021 than in 2019 and 2022 due to the control measures taken to fight SARS-COV-2 pandemic. Other respiratory infection showed a similar trend in the last 2–3 years in Guangzhou.^24^, ^25^, ^26^, ^27^. Olsen et al.^28^ reported the same trend in the United States of America (USA). In addition, competition among respiratory viruses or “virus interference” may have led to the dominance of SARS-CoV-2, reducing influenza infection.^8^

Our research showed seasonal fluctuations in influenza infection and incidence, and it is noteworthy that the peak at the end of 2021 did not exceed that in 2019. A study on seasonal influenza in China and the USA showed that influenza B virus declined sharply in China in early 2020, re-emerged in 2021 and reached a peak in 2022, which may account for the resurgence of infection and incidence in late 2021.^29^ Antigenic, genetic characteristics, and antiviral susceptibility of influenza viruses also supported the trend of re-emerge.^30^ Wang et al.^31^^,^^32^ also reported the increasing population susceptibility to seasonal influenza during the pandemic of coronavirus disease 2019 (COVID-19) in China. Both the 0–4 and 5–14 age groups were observed to have dramatically increased in spring and summer of 2022, which might be explained by the so called “immunological debt” theory. The population was more susceptible than before the pandemic of COVID-19. Our research showed that 67.89% and 65.02% of the 0–4 and 5–14 age groups were infected in 2022, which were over 11.5 and 4.5 times more than that of in 2021. For infants and children (0–14) and adults (> 14), 66.15% and 3.43% were infected in 2022, respectively. This was 5.9 and 9.8 times more than in 2021, 2.4 and 0.7 times more than in 2019, and 18.5 and 3.3 times more than in 2020. White et al.^33^ found that children and adolescents under 18 had high influenza incidence and disease severity in the USA in 2022–2023 influenza season. Therefore, the “immunological debt” theory might be valid. Non-pharmaceutical interventions (NPIs), such as social distancing measures and personal protective measures, taken to fight against the SARS-COV-2 pandemic effectively reduced the spreading of the respiratory viruses.^32^ The routine pattern of influenza epidemics was disrupted by the NPIs. Fewer people got infected by the influenza virus during 2020 to 2021, and the so-called “immunological debt” was accumulated. When reducing or ceasing the implementation of NPIs, people would be more susceptible to be infected on a population scale. Furthermore, adults possess a more developed immune system and have acquired partial immunity through repeated exposure to influenza viruses. By contrast, the less mature immune system of childhood lacks immunological defense against various influenza strains. Consequently, children exhibit higher susceptibility to influenza infection and a greater risk of morbidity upon exposure, as evidenced by statistical data.

We acknowledge several limitations to the current study. Firstly, analysis was dependent on data sources that may not accurately capture the true incidence of influenza. Surveillance systems frequently rely on reported cases and may be affected by healthcare-seeking behavior and access to medical services. Underreporting may result, particularly among populations with restricted healthcare access or in instances where individuals do not seek medical attention for mild symptoms, causing underestimation of incidence rates. Secondly, we employed multiplicative modeling to analyze influenza time-series data, a widely used method. However, formal statistical diagnostics were omitted. While results were consistent with prior research, this gap limits rigorous assessment of model fit. Future work should integrate advanced approaches such as generalized additive models and comprehensive diagnostic procedures to enhance predictive performance. Thirdly, the study coincided with the COVID-19 pandemic which may have affected influenza transmission dynamics. Anti-COVID-19 public health measures, such as social distancing and mask-wearing, may have reduced influenza spread and produced atypical incidence patterns. This confounding factor complicated the interpretation of influenza trends during the timeframe. Finally, demographic and geographic variations within Guangzhou have not been accounted for, reducing generalizability. Variations in population density, age distribution and vaccination coverage across districts may yield heterogeneous influenza incidence rates that are not captured by the current analysis.

In conclusion, infants and children were the main target of influenza and preventive measures should target this age group. Influenza vaccines are cost-effective and enhance herd immunity on a population scale, mitigating “immunological debt”. Surveillance in kindergartens, primary schools and middle schools should be strengthened, especially during influenza season. Parents of infants and school administrators should have easy access to epidemic information and targeted strategies for the prevention and control of influenza spread among infants and children should be implemented.

## CRediT authorship contribution statement

**Di Wu:** Writing – review & editing, Writing – original draft, Formal analysis, Data curation, Conceptualization. **Zhaonian Tan:** Writing – original draft, Formal analysis, Data curation. **Yanhui Liu:** Writing – review & editing, Supervision, Methodology, Funding acquisition, Data curation. **Mengmeng Ma:** Supervision, Software, Methodology, Investigation, Data curation. **Zhitao Chen:** Writing – review & editing, Supervision, Software, Resources, Formal analysis, Data curation. **Dedong Wang:** Writing – review & editing, Supervision, Software, Conceptualization. **Lei Luo:** Writing – review & editing, Validation, Supervision, Funding acquisition. **Pengzhe Qin:** Writing – review & editing, Writing – original draft, Supervision, Funding acquisition, Formal analysis, Conceptualization.

## Informed consent

This study was approved by the Ethics Committee of the Guangzhou Center for Disease Control and Prevention. Since the research is based on a de-identified public health surveillance program, the ethics committee exempted the requirement for obtaining individual informed consent. All data were fully anonymized before we accessed them for analysis.

## Organ donation

Not applicable.

## Ethical statement

This Study was approved by the Ethical Committee of Guangzhou Center for Disease Control and Prevention (Approval No.: GZCDC-2018018).

## Data availability statement

The datasets generated and/or analyzed during this study are not publicly available but can be obtained from the corresponding author upon reasonable request.

## Animal treatment

Not applicable.

## Generative AI

The authors declare that no generative AI was used in the creation of this manuscript.

## Funding

This work was supported by the 10.13039/501100003453Natural Science Foundation of Guangdong Province (2019A1515011407), Medical Science and Technology Project of Guangzhou (20201A011067, 20211A011059, 20241A011048), Guangdong Medical Science and Technology Research Project (A2019379, A2020399, B2021244). The 10.13039/501100015806Key Project of Medicine Discipline of Guangzhou (No. 2025–2027-11), Science and Technology Project of Guangzhou (202206080003, 2023A03J0457, 2025A03J3771).

## Declaration of competing interest

The authors declare that the research was conducted in the absence of any commercial or financial relationships that could be construed as a potential conflict of interest.

## Appendix Supplementary materials

Image, application 1
