# Supplementary material for: Estimated incidence of influenza in Guangzhou, China, 2019–2022
Source: Infect Med (Beijing). 2025 Nov 20;4(4):100221. doi: 10.1016/j.imj.2025.100221 (PMC12800747; doi:10.1016/j.imj.2025.100221)
Supplement: Supplementary file 1 [file mmc1.docx]

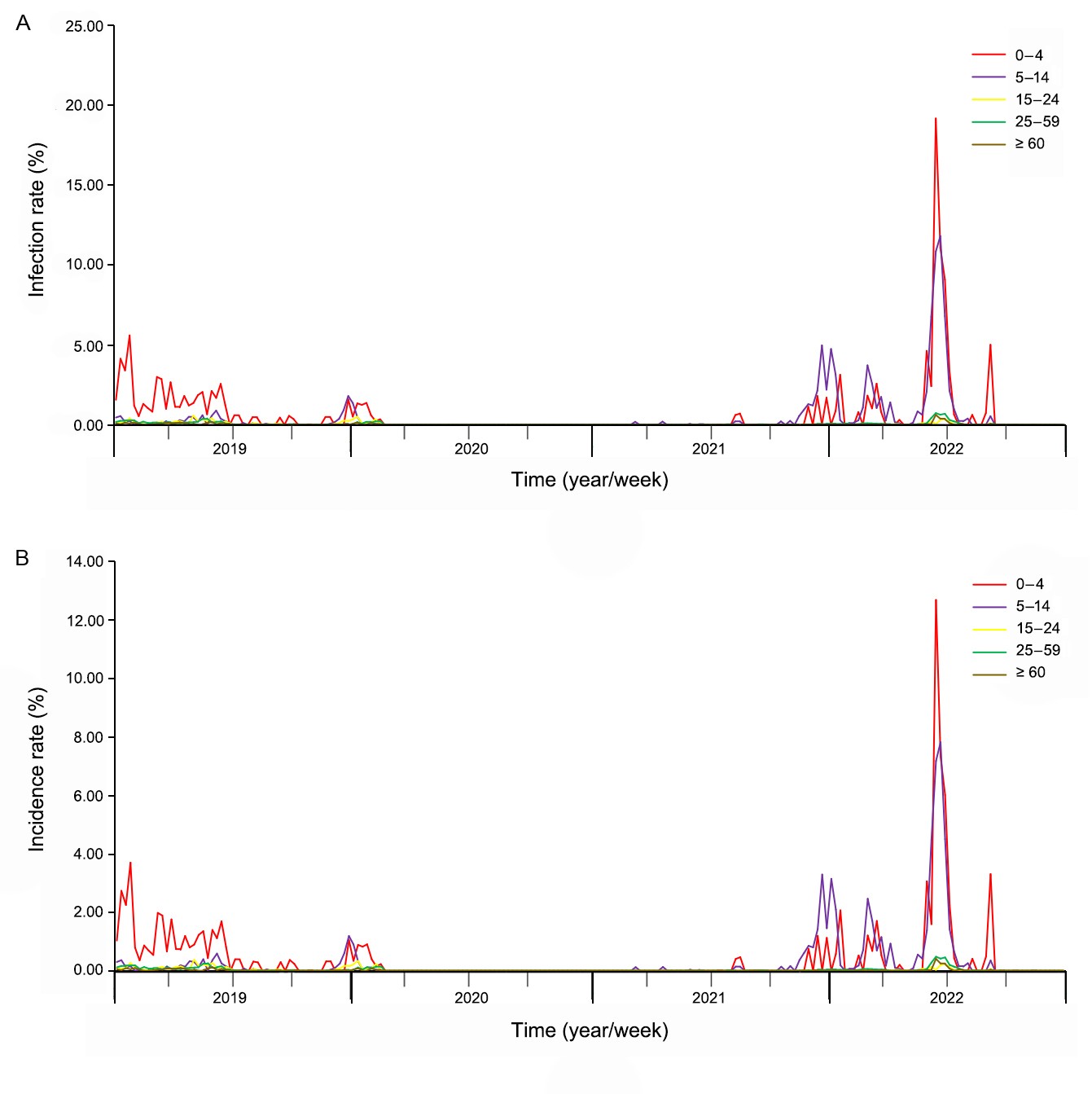


Supplementary Fig. S1. Weekly infection and incidence rates of Guangzhou 2019–2022. (A) Weekly infection rates of Guangzhou 2019–2022. (B) Weekly incidence rates of Guangzhou 2019–2022.
